# Do Social Support and Loneliness Influence Emerging Adults’ Mental Health during the First Year of the COVID-19 Pandemic?

**DOI:** 10.3390/brainsci13121691

**Published:** 2023-12-07

**Authors:** Lauri A. Jensen-Campbell, Angela Liegey Dougall, Abigail C. Heller, Priya Iyer-Eimerbrink, Michelle K. Bland, Kristen Hull

**Affiliations:** 1 Department of Psychology, University of Texas at Arlington, Arlington, TX 76019, USA; adougall@uta.edu (A.L.D.); bland.michelle@mayo.edu (M.K.B.); kristen.hull@mavs.uta.edu (K.H.); 2 Department of Psychology, Belmont University, Nashville, TN 37212, USA; abigail.heller@belmont.edu; 3 Department of Psychology, University of North Texas at Dallas, Dallas, TX 75241, USA; priya.eimerbrink@untdallas.edu

**Keywords:** social support, loneliness, emerging adults, COVID-19, pandemic

## Abstract

Youths’ mental health is at a crisis level, with mental health problems doubling in the US since the pandemic began. To compound the mental health crisis, there is a global loneliness epidemic, with emerging adults worldwide experiencing some of the highest rates. One study with two phases examined the influence of social support and loneliness on mental health in US emerging adults during the pandemic, including changes in these relationships over one year. Emerging adults (*N* = 449) completed online questionnaires via Prolific in May 2020 (Phase 1) and again from January to May 2021 (*N* = 253; Phase 2). More perceived support was related to reduced loneliness, with family support having the most significant influence. Loneliness mediated the link between perceived support and adverse health outcomes. Higher loneliness predicted more perceived stress and sleep difficulties concurrently and over time. There was a bidirectional relationship between loneliness and depression, such that higher levels of either variable at Time 1 predicted increases in the other over time. Results highlight the detrimental impact of loneliness on emerging adults’ mental health.

## 1. Introduction

“Loneliness is far more than just a bad feeling—it harms both individual and societal health. It is associated with a greater risk of cardiovascular disease, dementia, stroke, depression, anxiety, and premature death. The mortality impact of being socially disconnected is similar to that caused by smoking up to 15 cigarettes a day, and even greater than that associated with obesity and physical inactivity.”

(Office of the Surgeon General, 2023, p. 5) [1]

A Surgeon General’s report in the US is meant to make the population aware of an urgent public health issue. It is reserved for only those health challenges that are so concerning that they need immediate action. Of growing concern is how the current pandemic has further impacted the mental health of children and emerging adults, which was already at crisis levels before the pandemic [2]. Increasing evidence suggests that psychosocial factors associated with the pandemic, such as social distancing and increased loneliness, exacerbated mental health issues in youth worldwide, with depression and anxiety doubling in the United States since the pandemic began [3]. Even before the 2020 pandemic, the United States and many countries worldwide have been on the verge of a loneliness epidemic [4]. Since the pandemic began, not only has the number of people who report profound loneliness increased, but also the frequency of their loneliness, with lower age (i.e., emerging adulthood) as a risk factor [5,6,7].

Indeed, loneliness is now considered a serious public health concern worldwide [1,8,9]. The number of adolescents with elevated loneliness nearly doubled worldwide from 2012 to 2018, with girls seeing more significant increases [9]. Emerging adults globally have also shown increases in loneliness over the last 43 years [8]. The US often has some of the highest rates of loneliness. By region, adolescent loneliness increases were most prominent in English-speaking countries (e.g., UK, USA, and Canada) and orthodox countries, such as Russia, Bulgaria, and Central/South American countries [9]. Similarly, older adults in the US, England, Poland, and Spain report some of the highest levels of loneliness, ranging from 9% to 19% [10].

Loneliness is different from physical isolation, as people may feel lonely even in the presence of others [11]. Instead, loneliness is a distressing feeling that arises from dissatisfaction with social relationships—be it the quality, the quantity, or even a perceived discrepancy between the person’s desired interactions versus the reality [12]. Loneliness is so concerning because it is a severe health risk. Loneliness is associated with internalizing problems such as depression, anxiety, and suicidal ideation [13,14,15]. Additionally, loneliness is related to poorer sleep quality, less physical activity, increased cardiovascular risk, immune dysregulation, and even mortality [16,17,18,19].

This study involved two phases examining the influence of social support and loneliness on mental health outcomes in US emerging adults during the pandemic. The second phase, a follow-up longitudinal wave, used cross-lagged analyses to investigate changes in emerging adults’ loneliness, social support, and mental health problems. We chose the developmental period of emerging adulthood because recent research has found that nearly 61% of 18 to 25-year-olds report experiencing profound loneliness [7].

### 1.1. Emerging Adulthood and Loneliness

Distinct from adolescence and young adulthood, emerging adulthood is a developmental period encompassing the late teens and the mid-to-late twenties, with a particular interest in ages 18 to 25 [20]. This is when individuals no longer feel like a child but do not believe they are adults (i.e., feeling “in-between”) [21]. Parents also do not view their emerging adult child as an adult yet [22]. This period is marked by many environmental changes and individual development, partly due to the rise of post-secondary education and a delay in commitments related to jobs and marriage [23]. During this developmental phase, individuals explore their identities more intensely, experience more instability, and focus more on themselves [21].

Because emerging adulthood is a period with a propensity for change and growth, it may put these children at greater risk for problems such as loneliness if these opportunities for change and growth are thwarted. Children in late adolescence and emerging adulthood often feel lonelier than individuals in other age groups, except those in very old age [24,25,26,27]. Indeed, loneliness follows a U-shaped curve, peaking in both emerging and late adulthood [26,28,29]. Even more concerning is the finding that loneliness rates among this age group have increased steadily since the 1970s [8].

Emerging adulthood is also a developmental period during which individuals may be especially vulnerable to stressors. Most psychiatric disorders develop by age 24, with the onset ranging from around 15 years and peaking in the early 20s [30,31]. The onset of disorders during this time is especially alarming, considering how mental disorders may hinder the personal and professional growth of emerging adults [31]. The development of psychopathologies may be exacerbated by events specific to the time of life, as young adults often experience academic and professional stress about beginning college and making decisions about their careers [32].

The circumstances of the COVID-19 pandemic can change the trajectories of many emerging adults. First, social distancing limits their autonomy and freedom to explore their identity entirely. Social bonds are increasingly important during this time, and social distancing measures interrupt the development of these bonds, isolating emerging adults from family and friends. Longitudinal research of multiple generations showed that younger cohorts tend to experience more loneliness, indicating that the impact of social isolation and loneliness could be experienced more intensely by these emerging adults [33]. Indeed, emerging adults suffer from some of the highest rates of loneliness worldwide [1,34,35,36,37].

### 1.2. Loneliness and Mental Health

Understanding loneliness in emerging adults is critical because it often exacerbates certain psychopathologies, including the ones examined in the current study [38,39,40]. Loneliness is distinct from depression and anxiety, and loneliness may be responsible for increasing these internalizing problems. Since the first outbreaks of COVID-19, psychosocial responses to the pandemic have been highly negative [41,42], persistent over time [42,43,44], and strongly associated with reported loneliness [45,46,47] and social isolation [48]. Reports from the early stages of the pandemic in mid-2020 suggest that emerging adults are at higher risk of loneliness [34,36,37] and experience disruptions to psychosocial health and well-being related to loneliness [15,46,49]. Thus, in the current study, we sought to examine loneliness among emerging adults in the first year of the pandemic and collected key internalizing problems (i.e., depression, PTSD symptoms, stress, sleep difficulties) during the two waves.

#### 1.2.1. Depression

Though loneliness and depression are distinct constructs, they are related [38,39]. In a meta-analysis of 88 studies on loneliness and depression, Erzen and Cikrikci (2018) found that loneliness had an overall moderate effect on levels of depression [50]. The relationship between loneliness and depression can be seen in multiple populations; for example, Murata and colleagues (2021) found that loneliness predicted higher depressive symptoms for adolescents, adults, and healthcare workers during the pandemic [13]. In a three-year longitudinal study of adults aged 50 to 67, Cacioppo and colleagues (2006) found reciprocal influences between loneliness and depressive symptoms [51]. Similarly, early peer-related loneliness has been shown to predict higher depressive symptoms in adolescence [52] and middle and later adulthood [53]. Because of the documented relationship between loneliness and depression, we expected loneliness to predict increases in depression over time.

#### 1.2.2. Post-Traumatic Stress Symptoms (PTSS)

Loneliness can also lead to symptoms associated with post-traumatic stress disorder (PTSD), even years after a trauma has occurred. For example, Shevlin and colleagues (2015) found that loneliness mediated the relationship between childhood trauma and adult PTSD [54]. More childhood trauma predicted higher levels of loneliness, which then predicted higher levels of PTSD in adults. According to the American Psychiatric Association (2013), symptoms associated with post-traumatic stress include experiencing sleep disturbances, negative mood alterations, emotional reactivity, and frightening dreams [55]. There is a well-documented trend that increases in subclinical PTSD symptoms or PTSS, which are associated with modern large-scale pandemics [56], such as SARS (2003) [57,58], influenza (2009) [59,60], Ebola virus (2014) [61,62], and MERS (2015) [63,64]. In a study of adolescents, adults, and healthcare workers during the 2020 COVID-19 pandemic, Murata and colleagues (2021) found that loneliness was the most common predictor across multiple psychological outcomes, including PTSS, in all sample groups [64]. Additionally, Kalaitzaki and colleagues (2022) found that higher levels of loneliness were associated with higher PTSS in Greece [65]. As such, we hypothesized that loneliness would predict increases in COVID-related post-traumatic stress symptoms (CPTSS) over time.

#### 1.2.3. Stress

Loneliness also strongly correlates with physiological and reported stress [40]. Higher loneliness is associated with increased hypothalamic–pituitary–adrenal axis (HPA) activity (implicated in reactions to stress and the release of cortisol) and lowered immunity, subjective well-being, and executive functioning [40,66]. Thus, lonely people tend to experience higher rates of inflammation and morbidities [67,68] and have an increased mortality rate [69] compared to less lonely controls. In a longitudinal study of adolescents aged 15 to 20, Vanhalst and colleagues (2013) found that individuals who reported lower levels of loneliness throughout the five years of the study had the best psychosocial functioning, while those who experienced chronic loneliness exhibited the worst psychosocial functioning (e.g., higher perceived stress, depression, and anxiety) [70]. Contextualized with COVID-19, early studies demonstrated that levels of both perceived stress [71] and loneliness [46] reported by emerging adults during the pandemic significantly exceeded pre-pandemic levels; thus, in the current study, we expected loneliness to predict increases in perceived stress over time.

#### 1.2.4. Sleep Difficulties

The effects of loneliness are felt even while we sleep. Kurina and colleagues (2011) found loneliness to correspond with a direct increase in sleep fragmentation [72]. Similar studies examining sleep report links between loneliness and poorer sleep quality [73] and increased daytime dysfunction [74]. Conversely, individuals with higher support report better sleep quality [75]. More recently, in a propensity-score-matched case–control study in China, lonely people had longer sleep latencies, woke up more frequently at night, reported poorer subjective sleep, and were more fatigued during the day than their non-lonely peers [19]. Lonelier individuals experience less restorative sleep and often feel drained of energy, lacking the physical and emotional resources to cope with other stressors or any pre-existing health problems, with lonely individuals being 2.67 times more likely to sleep less than usual while also being 1.92 times more likely to sleep more than usual during the beginning of the pandemic [19,75,76,77].

Furthermore, both energy level and sleep dysfunction have been separately correlated with loneliness [72,73,74], and those who experience social isolation tend to exhibit poorer sleep habits and insomnia [78]. Emerging adults who experience higher levels of loneliness are significantly more likely to report inferior sleep quality, reduced sleep efficiency, longer wake time before sleep onset, and more significant daytime dysfunction [79,80]. Given the established connection between loneliness and sleep, we hypothesized loneliness to predict increases in sleep difficulties over time.

#### 1.2.5. Perceived Social Support

Social support is vital in reducing loneliness across all age groups and reducing vulnerability to adverse health effects associated with loneliness, such as stress and related health symptoms. It is crucial during significant developmental changes, as in emerging adulthood [81]. Generally, social support is suggested to reduce loneliness, improve quality of life, increase subjective well-being, and decrease depression and hopelessness in vulnerable groups, such as older adults [82,83,84]. Following traumatic events, research shows strengthened support from one’s social networks to moderate the relationship between anxiety and depression [85]. Social support also buffers against stress and its associated symptoms, such as depression, and improves overall health by reducing loneliness [86,87].

Though emerging adults were among the loneliest during the pandemic across age distributions [34,35,36,37,88], increases in social support during COVID-19 have predicted lower levels of loneliness among samples of college students [89,90]. Reductions in face-to-face interactions due to social distancing protocols in 2020 and 2021 saw increased online interactions and social media usage in this group [91,92]. Interestingly, higher rates of online social interaction elicit perceptions of social support among emerging adults without physical connection [93] and mediate the relationship between the age group and loneliness [91].

During emerging adulthood, the nature of relationships also changes; most emerging adults report having close friends and romantic partners. These relationships are distinct and can provide different patterns of support [94,95]. For example, friends provided higher levels of companionship and intimacy, while parents provided more affection and instrumental help [96]. Both friend and family support have been linked to maintained or improved mental health during adjustments to college [97]. Friends, however, are often rated higher than parents for overall support [96], and the relationship between friend support and loneliness is more robust than it is for either family or significant other support [81,84].

Emerging adults spend increasingly more time with their friends and romantic partners; the positive qualities of both relationships are related to less loneliness [98]. However, these relationships are voluntary and can even be transient, given that emerging adults can enter and leave these relationships freely [99,100]. Even though emerging adults rely more on their friends, parental support is still essential to young adults. Indeed, emerging adults still view parental support as valuable [101].

It is possible that when a friend’s and romantic partner’s support is limited, such as in a pandemic, parents’ support will be critical. Based on the abovementioned information, we expected social support’s buffering role on loneliness to be significant for each relationship type. That is, we expected all three sources of support to be associated with less loneliness (Phase 1). Given the transient nature of the support from friends and significant others, we also predicted that parental support might be critical during the pandemic. That is, lockdowns can limit the companionship that support from friends provides. Additionally, relationships with significant others may be strained by a lack of face-to-face interactions. Furthermore, we expected higher levels of social support at Time 1 to predict decreases in loneliness at Time 2.

#### 1.2.6. Loneliness as a Mediator

Given the findings from previous research, this study will also examine whether loneliness mediates the associations between perceived social support and mental health. It was expected that perceived social support would influence feelings of loneliness. Loneliness, in turn, would influence the mental health of emerging adults. Loneliness has been found to mediate the link between social support and subjective well-being [102,103]. Latent growth curve mediation models using the Longitudinal Aging Study Amsterdam also found that social support was associated with being less lonely. In turn, decreased loneliness was related to enhanced cognitive performance [104]. During the early months of the pandemic, loneliness even mediated the relationship between social support and elevated hope in samples from the UK, USA, and Israel [105]. Finally, loneliness mediated the relationship between family support and depressive symptoms among emerging LGBQ adults who were living with their parents during the pandemic [106]. As such, we wanted to examine whether loneliness mediated the associations between social support and mental health during the first year of the pandemic.

## 2. Materials and Methods

The current study involved a USA national sample of emerging adults to assess the influence of loneliness and social support on mental health. The first phase involved a sample from Prolific at the start of the pandemic. Prolific (http://www.prolific.com) is a UK-based research platform that connects researchers to potential research participants around the globe. We solicited potential participants who currently lived in the USA aged 18 to 25 years.

Phase 1 was a single online session using Qualtrics, a survey-collection platform. Following informed consent, those who agreed to participate completed a series of online surveys that took approximately 30 min, measuring loneliness, stress, and health outcomes. Demographic information was also collected. Participants were told that only individuals correctly answering the attention checks would receive compensation. We further said to them that attention checks were included in the survey to ensure that instructions were followed, and attention was paid to the questions being asked. Once participants completed the study, they were thanked for participating, paid USD 4.00 if they passed the attention checks, and provided with information about crisis resources.

Phase 1 examined whether loneliness would mediate the relationship between social support and mental health. We expected that social support would be linked to less loneliness, which would be related to less stress, depression, PTSS, and sleep problems. We also examined the alternative model where social support mediates the relationship between loneliness and mental health, given that some studies have examined this alternative model (e.g., Hutten et al., 2021) [107]. Three specific sources of support were examined: family, friends, and significant others. We anticipated that family support would be vital when other sources of support are limited.

The second phase involved a follow-up wave approximately a year later. For Phase 2, participants in Prolific completed identical surveys nine months later, from 11 January 2021 to 12 May 2021. Once participants completed the survey, they were thanked for participating, paid USD 4.75, and given information about crisis resources. Phase 2 allowed us to more carefully examine whether social support would be associated with decreased loneliness during the first year of the pandemic. We did not think loneliness would influence perceived support over time. Finally, the two phases allowed us to examine whether loneliness was related to increased psychological difficulties over the first year of the pandemic [46,70]. We did not expect internalizing problems to be related to increases in loneliness. However, we examined the competing hypotheses that mental health influences change in loneliness. The University of Texas at Arlington IRB approved this study (2020-0633).

### 2.1. Participants

A total of 449 participants aged 18 to 25 participated in Phase 1 (M = 23.26, SD = 2.31) for an online survey with compensation. Participants completed Phase 1 from 21 May to 28 May 2020, when deaths in the United States related to COVID-19 had risen to approximately 1000 deaths per day [108]. During this time (March to May 2020), 42 states and territories issued mandatory stay-at-home orders, which affected 73% of US counties [109]. 

There were 225 males, 212 females, 11 nonbinary, and 1 participant who preferred not to report a gender within the sample. Most of the sample identified as non-Hispanic White (*N* = 147, 32.7%), followed by Asian (*N* = 130, 28.9%), Hispanic/Latino (*N* = 89, 20.0%), Black (*N* = 46, 10.2%), and Multiracial/other (*N* = 30, 6.6%). Four participants declined to provide ethnic and race information. Most of the sample (*N* = 442) had completed high school education, with nearly half (*N* = 199, 44.3%) having completed a university or post-graduate degree.

For Phase 2, 253 emerging adults from Prolific (111 Male, 134 Female, and 8 nonbinary) completed the survey again from 11 January 2021 to 12 May 2021. At this time, all stay-at-home orders had been lifted since August 2020. Of the 449 who completed Phase 1 in Prolific, 291 (65%) remained active on Prolific in 2021. A total of 86.9% of these active users (56.8% of the total baseline sample) were part of the final sample and received USD 4.75 for participating. The longitudinal sample, including both phases, was still diverse, including 53 Latinos, 81 non-Hispanic Whites, 25 Blacks, 71 Asians, and 20 participants who identified as Biracial/other.

Given that 43.2% of the Prolific sample did not complete the second phase, analyses were run to examine possible differences between the groups. Participants only differed on gender; female and non-binary participants were more likely to continue participating (χ^2^(2) = 9.76, *p* = 0.008), similar to the findings of other studies [110]. There were no differences in age, immigrant status, employment status, ethnicity, race, or education level. Additionally, when controlling for family-wise error rates, no differences existed on any of our focal variables at Time 1 (*p* < 0.001).

### 2.2. Materials

#### 2.2.1. Loneliness

Participants completed the UCLA Loneliness Scale-Version 3 [111], which measures loneliness and social isolation. Participants indicated how frequently they felt lonely on a 3-point Likert scale from 1 (“Hardly ever”) to 3 (“Often”) for each of the 20 items. Reliability was acceptable, α = 0.873, and ω = 0.872.

#### 2.2.2. Perceived Stress

The 10-item Perceived Stress Scale (α = 0.81, ω = 0.85) measured an individual’s perception of stress in the last month [112]. Participants rated how often they have felt stressed on a 5-point Likert scale from 1 (“Never”) to 5 (“Very Often”).

#### 2.2.3. Social Support

The 11-item Multidimensional Scale of Perceived Social Support was modified to assess social support during the COVID-19 pandemic [113]. Participants read statements (e.g., “Even while social distancing, there is a special person I can contact when I am in need.”) and rated their level of agreement from 1 (“Strongly disagree”) to 5 (“Strongly agree”). The MSPSS measured support from three sources: family (α = 0.829, ω = 0.837), friends (α = 0.824, ω = 0.828), and significant other (α = 0.90, ω = 0.90).

#### 2.2.4. CPTSS

The Short PTSD Inventory (α = 0.936, ω = 0.936) was modified to evaluate PTSD symptomatology related to the COVID-19 pandemic (CPTSS) [114]. Participants read a total of 8 statements and then indicated how strongly the statement applied to themselves from 1 (“Not at all”) to 5 (“Extremely”).

#### 2.2.5. Depressive Symptomatology

The 10-item Center for Epidemiologic Studies Depression Scale (CES-D; α = 0.869, ω = 0.876) assessed how often emerging adults experienced depressive feelings and behaviors over the past week [115]. Participants indicated how often they felt depressed during the past week from 0 (“Rarely or none of the time/less than one day”) to 3 (“Most or all of the time/5–7 days”).

#### 2.2.6. Sleep Difficulty

Participants completed a modified Pittsburgh Sleep Quality Index (PSQI) [116]. For this study, we focused on the items that comprised Component 5, which involved sleep difficulties. Items were rated from 1 (‘not during the past month”) to 4 (“three or more times a week”). As part of Component 5, we added two questions: “Have nightmares” and “Cannot stop thinking about COVID-19.” We then summed the 11 items involving sleeping difficulty (ω = 0.84, α = 0.841).

## 3. Results

### 3.1. Missing Data

Only participants who completed the survey and passed the attention check threshold were retained for Phase 1. Four attention checks were dispersed throughout the survey to mitigate the effects of poor-quality data from online sources. Participants who failed two of the three multiple-choice checks (improbable response) or provided unusual comments to an open-ended attention-check question were excluded from the final sample. Unusual responses included single words that did not align with the question (e.g., good, nice) or nonsensical phrases or answers that had nothing to do with the question [117]. For Prolific, 449 of the original 465 participants were retained and paid (97%).

A missing value analysis (MVA) was then performed on the final sample using SPSS. The results indicated that no variable had more than 5% of values missing, and missing data were missing completely at random (MCAR).

### 3.2. Does Social Support Alleviate Loneliness?

We used hierarchical regression to enter gender, education, and age as control variables. Since gender involved three groups, gender was coded into two variables using indicator codes (females vs. males, females vs. non-binary/other). Family, friends, and significant other (SO) social support were used as predictors in the second step. Regression analyses were supplemented with dominance analysis (DA) to determine whether certain types of support had a more significant influence on loneliness [118].

Women reported more loneliness than men (*b* = −0.18, *t*(440) = −3.32, *p* < 0.001, and *sr^2^* = 0.024). There was no difference between women and non-binary participants on loneliness (*b* = −0.15, *t*(440) = −0.82, *p* = 0.41, and *sr^2^* = 0.001). Neither age nor education predicted loneliness. The three sources of social support predicted loneliness over and above our control variables (Δ*F*(3, 440) = 50.49, *p* < 0.011, and Δ*R^2^* = 0.276). Family support was negatively associated with loneliness (*b* = −0.14, *t*(440) = −3.57, *p* < 0.001, and *sr^2^* = 0.059). Emerging adults with higher SO support also reported less loneliness (*b* = −0.10, *t*(440) = −3.58, *p* < 0.001, and sr^2^ = 0.02). Finally, higher friend support was associated with less loneliness (*b* = −0.10, *t*(440) = −3.38, *p* < 0.001, and sr^2^ = 0.019).

Next, DA using R 4.2.1 evaluated the explanatory power of each type of social support alone and with all possible combinations of predictors to determine the relative importance of each type of support [118]. Dominance analysis permitted us to ask whether certain types of social support contributed more variance to the regression effect for models containing all or some subsets of our predictors. Control variables and social support were entered into the model as predictors. All forms of social support had higher dominance than any of our control variables. Family support contributed the most to decreased loneliness (0.103), followed by significant other (0.071) and friend support (0.072). Family support was more dominant in the complete, conditional, and general DA than friends’ or SO support. Additionally, SO support and friends’ support were not different from each other. Put another way, family support had a higher unique variance contribution to loneliness than friends’ or SO support in all sub-models.

### 3.3. Is Loneliness a Mediator between Social Support and Health Outcomes?

Using SEM, a single mediation model in lavaan (R 4.2.1) was run to examine the possible mediating influences of loneliness on the support-psychological problem relationship. We also examined the direct effects between social support and health outcomes, which gave us a saturated model. Our endogenous variables were family, friends, and SO support, with our controls of gender, education, and age. Our mediator was loneliness. Outcome variables included in the single model were (1) depression, (2) CPTSS, (3) perceived stress, and (4) sleep difficulties (see Table 1 and Table 2). Women reported being lonelier than men (*b* = −0.20, *SE* = 0.05, *z* = −4.24, and *p* < 0.001). There were no differences between nonbinary participants and women and men (*b* = −0.10, 0.10; *SE*s = 0.15; *z* = −0.67, 0.67; *p*s = 0.50). Social support and the control variables account for 27.5% of loneliness variance.

#### 3.3.1. Depression

The model accounted for 37.8% of the variance in depression. Loneliness was positively related to depression (*b* = 0.46, *SE* = 0.04, *z* = 11.12, and *p* < 0.001). Those emerging adults with more family support reported lower levels of depression (See Table 1 and Table 2). Men reported less depression than women (*b* = −0.16, *SE* = 0.04, *z* = −3.63, and *p* < 0.001). Nonbinary participants also reported more depression than men (*b* = 0.30, *SE* = 0.13, *z* = 2.30, and *p* = 0.02). There were no differences between women and nonbinary participants (*b* = 0.14, *SE* = 0.13, *z* = 1.10, and *p* = 0.27). Neither friend nor significant other support directly influenced depression (See Table 1). However, all three sources of social support were indirectly related to depression via loneliness (See Table 2). Participants with more support reported less loneliness, which was related to lower levels of depression. In sum, parent support influenced depression directly and via decreases in loneliness. Friends’ and significant other’s support buffered against depression via decreasing loneliness.

#### 3.3.2. CPTSS

The mediation model accounted for 5.1% of CPTSS symptoms. Greater loneliness predicted more CPTSS (*b* = 0.28, *SE* = 0.09, *z* = 3.29, and *p* < 0.001). Contrary to expectations, friend support was positively related to CPTSS. All three sources of social support were negatively associated with CPTSS via their negative relationship with loneliness (See Table 2).

#### 3.3.3. Perceived Stress

The mediation model accounted for 37.4% of perceived stress. As anticipated, loneliness was positively related to perceived stress (*b* = 0.56, *SE* = 0.06, *t* = 10.06, and *p* < 0.001). Emerging adults with more family support were less stressed. Contrary to expectations, friend support was positively related to perceived stress. Family, friends, and significant other support were also indirectly related to stress, such that lacking support led to being lonelier and more stressed (See Table 1 and Table 2). Older emerging adults also reported more stress (*b* = 0.03, *SE* = 0.015, *z* = 1.96, and *p* = 0.05). Men reported less perceived stress than women (*b* = −0.29, *SE* = 0.057, *z* = −5.00, and *p* < 0.001). Nonbinary participants reported more perceived stress than men (*b* = 0.46, *SE* = 0.17, *z* = 2.64, and *p* = 0.008). There were no differences between women and nonbinary participants (*b* = 0.17, *SE* = 0.17, *z* = 0.99, and *p* = 0.32).

#### 3.3.4. Sleep Difficulties

The model accounted for 14.7% of sleep difficulties. Loneliness was again related to more sleep problems (*b* = 0.35, *SE* = 0.05, *z* = 7.22, and *p* < 0.001). All three sources of support were related to fewer sleep problems via loneliness, such that support led to decreases in loneliness, which in turn led to fewer sleep difficulties. Men were less likely to have sleep difficulties than women (*b* = −0.10, *SE* = 0.05, *z* = −1.91, and *p* = 0.056). Additionally, nonbinary participants reported more sleep difficulties than men (*b* = 0.34, *SE* = 0.15, *z* = 2.20, and *p* = 0.028). There were no differences between women and nonbinary participants (*b* = 0.24, *SE* = 0.15, *z* = 1.57, and *p* = 0.12).

### 3.4. Alternative Mediator Model

Finally, we compared the “loneliness as a mediator” to the alternative “social support as a mediator” model. We began by setting the direct paths from social support to mental health to 0, creating an unsaturated model. The overall model fit of our “loneliness as a mediator” is acceptable (CFI = 0.97, RMSEA = 0.086, and SRMR = 0.03). This model provides additional evidence that the influence of social support on mental health is mediated via loneliness.

When examining whether the alternative model that loneliness predicted mental health via social support, we began by setting the direct paths from loneliness to mental health to 0, creating an unsaturated model. This model did not fit the data well (CFI = 0.92, RMSEA = 0.26, and SRMR = 0.06).

Finally, we used the Akaike Information Criterion (AIC) and Bayesian Information Criterion (BIC) to compare our two saturated models. The BIC and AIC are comparative fit indices for contrasting competing non-nested models and can be used with saturated models. There is no cut-off or acceptable value for AIC and BIC, but they are used to compare models with the lowest AIC and BIC, offering the best fit [119]. The “loneliness as a mediator” model had a better fit (AIC = 6746.76; BIC = 6990.72) than the alternative “social support as mediators” model (AIC = 6915.88; BIC = 7194.09).

### 3.5. Does Social Support Lead to Decreased Loneliness?

To further examine the directionality of the social support–loneliness relationship, three cross-lagged panel analyses using lavaan in R 4.2.1 examined whether support at Time 1 (T1) predicted less loneliness at Time 2 (T2). Additionally, this model simultaneously examined whether loneliness influenced changes in support. Gender (female vs. male; female vs. nonbinary), Age, and Education were entered into each model as control variables. Interestingly, greater T1 perceived family and SO support predicted decreased loneliness at T2, but not vice versa (See Table 3 for the results). Friend support was not related to decreased loneliness over time but was still related to loneliness concurrently at T2 (*r* = −30, *p* < 0.001). Overall, these analyses support the notion that more support can reduce loneliness. However, we found no evidence that loneliness influenced perceptions of support over time.

It should be noted that there were seven cross-lagged models tested in 3.5 and 3.6. When correcting for possible family-wise error rates, the new critical *p*-value would be 0.007. Using this *p*-value, none of our support measures were related to changes in loneliness over time. However, all support measures were related to lower levels of loneliness at each time point.

### 3.6. Does Loneliness Predict Changes in Mental Health?

Next, we examined whether greater loneliness led to increased psychological problems over time in four cross-lagged panel analyses using lavaan in R 4.2.1. Gender, age, and education were again entered into each model as control variables. As anticipated, greater loneliness (T1) predicted increased stress, CPTSS, and sleep difficulties at T2 (See Table 4). A bi-directional effect was found between loneliness and depression. That is, loneliness is both a predictor of and predicted by depression. Women also reported more increased sleep difficulties than men, *b* = −0.15, *SE* = 0.06, *z* = −2.82, *p* = 0.004. Overall, these analyses supported the notion that loneliness can lead to more psychological problems and mediate the link between support and outcomes.

## 4. Discussion

A growing concern is how the current pandemic has influenced emerging adults’ loneliness and mental health. Even before the events of the 2020 pandemic, the world had been on the verge of a loneliness epidemic, especially for emerging adults [4]. Since the pandemic began, not only has the number of emerging adults who report profound loneliness increased, but also the frequency at which they feel lonely [7]. The current study is unique in examining the influence of social support and loneliness on mental health throughout the first year of the COVID-19 pandemic for a developmental cohort more susceptible to loneliness and the emergence of mental health problems.

The study’s first phase examined whether social support influenced loneliness at the start of the pandemic. We hypothesized that support during the pandemic was expected to be related to less loneliness. Although we did not have specific predictions about how our three sources of support might differentially influence loneliness, examining possible differences can provide insight into these essential sources of support during times of heightened difficulty.

### 4.1. Social Support during the Lockdown

In Phase 1, more social support from family, friends, and a significant other was related to reduced loneliness, with family support having the most significant influence. The perceptions of social support from family and significant others, but not from friends, were also associated with decreased loneliness. Given that loneliness did not predict changes in social support, this study provides further evidence that perceived support from others is critical to reducing feelings of loneliness.

Before the COVID-19 pandemic, help from friends appeared to be more salient for this age group and more effective in reducing loneliness than other sources of support [120]. From 2020 to 2021, disruptions in social support due to COVID-19 restrictions were likely to have been particularly salient as individuals of all ages reduced social contact with friends in adherence to preventative health recommendations [121]. For young people, friends were then presumably harder to see in person than family members or significant others; interestingly, within this context, remote or virtual interactions were not associated with lower loneliness, whereas in-person interactions were [122]. Other findings across the general population during COVID-19 outbreaks further specified that the availability of social support [36], as well as the number of close friends [34], were more strongly associated with a decrease in loneliness, instead of perceptions of support received from these individuals. Hajek and Konig (2021) found that less frequent internet contact with friends and family during the pandemic predicted higher loneliness among German adults who were forty years or older [123]. In reference to collective results found in both phases, difficulties in maintaining good contact with friends, specifically, are likely to have reduced the efficacy of this social support source in context, resulting in the disproportionate changes in loneliness seen across time among our sample of emerging adults. This study also highlights the continued importance of family support, especially during a period of difficulty such as a pandemic.

### 4.2. Loneliness and Mental Health

Next, this study examined whether loneliness mediated the relationship between social support and mental health. The longitudinal data further explored the directionality of our mediation model. In other words, the addition of the second phase allowed us to examine whether loneliness predicted changes in mental health difficulties or whether these difficulties influenced loneliness. Mental health measures included depression, CPTSS, perceived stress, and sleep difficulties.

### 4.3. Depression

Social support was related to depression via loneliness at Time 1 and predicted increases in depression over time. The results coincide with previous research suggesting that loneliness predicts depression concurrently [50] and over time [51,52,124]. Interestingly, depression also predicted increases in loneliness over time. These findings overlap with Cacioppo and colleagues (2006), who found a bidirectional relationship between loneliness and depression over time [13]. More recently, Fincham and May (2023) found a bidirectional relationship between loneliness and depressive symptoms over time in emerging adults during the pandemic [125].

### 4.4. COVID-Related PTSS (CPTSS)

Emerging adults’ reported loneliness did mediate the line between social support and CPTSS at Time 1. Additionally, loneliness was related to changes in CPTSS over time. The results matched previous research pointing to loneliness as a concurrent predictor of PTSS during the early days of the COVID-19 pandemic [43,126]. Kalaitzaki et al. (2022) also found that loneliness predicted COVID-related post-traumatic stress symptoms in two cross-sectional samples in Greece [65]. Because loneliness predicted increases in CPTSS over time, it could be that the pandemic also acted as more of a traumatic experience. Indeed, the negative impact of trauma increases as traumatic experiences become compounded over time [127], which coincides with current research claiming that loneliness predicted PTSS throughout the pandemic [128].

### 4.5. Perceived Stress

Higher levels of loneliness predicted higher levels of perceived stress and mediated the association between social support and stress. Loneliness was also related to increases in stress over time. The results support previous findings that examined perceived stress and loneliness among emerging adults during the first wave of COVID-19 outbreaks in early 2020 [129,130]. In comparison to other longitudinal study designs, young adults’ perceptions of stress appear to have increased over time from both before the pandemic to the first wave of the pandemic in the spring of 2020 [71,131] and from the first wave to the second wave in fall 2020 [132].

Studies have shown that perceived stress is most significant among younger adults during the pandemic compared to other ages [133,134]. It should be noted that, as a group, emerging adults were disproportionally affected by COVID-19-related factors (e.g., school closures, shifts to remote learning, disproportionate job loss among this age group, and fear of COVID-19), which could have exacerbated this apparent stress vulnerability, therefore giving rise to the loneliness–stress relationship seen in the present study. Keeping these situational and contextual factors in mind, researchers should focus future efforts on examining the potential contribution of pandemic-specific stressors to perceived stress ratings.

### 4.6. Sleep Difficulties

Higher levels of loneliness also predicted more reported sleep difficulties. Similarly, loneliness predicted increases in sleep difficulties over time. The current study’s results correspond to similar studies in which higher levels of loneliness were associated with poorer sleep quality in emerging adults during the COVID-19 pandemic [135]. According to Perez and colleagues (2022), the reasons for the relationship between loneliness and sleep difficulties may include those related to inflammation. Loneliness is associated with higher levels of inflammation, which can change sleep-related neurotransmitters [136].

The results of the present study regarding the relationship between loneliness and sleep difficulties in emerging adults have implications for other health outcomes. For example, sleep disruption is associated with short-term effects such as somatic pain, lower quality of life, mood disorders, and reduced cognitive functioning, as well as with long-term outcomes such as hypertension and obesity [137]. Sleep difficulties are also related to reduced immune [138] and psychosocial [137] functioning. Evidence that loneliness has predicted increased sleep difficulties in emerging adults during the pandemic highlights the importance of targeting psychosocial factors such as loneliness in future research on sleep-related intervention efforts.

## 5. Conclusions and Future Directions

While this paper provides insight into the influence of emerging adults’ social support during the first year of the COVID-19 pandemic, some limitations exist. The sample consisted of emerging adults in the United States specifically. As a result, our findings may not translate to the loneliness and mental health outcomes of emerging adults living in other countries and among different cultures, who may face different struggles and considerations during the pandemic. For example, the protective influence of social support on loneliness is more robust in rural populations than in urban populations among Chinese samples [84]. Additionally, in a systematic review and meta-analysis examining the impact of the COVID-19 pandemic on the mental health of college students, Li and colleagues (2021) found that, when compared to Chinese students, non-Chinese students (including those in the United States) reported higher levels of depression and anxiety [139].

Additionally, it should be noted that other important risk factors predict both loneliness and psychological difficulties (e.g., prior history of depression, neuroticism, smoking, drinking, physical activity, etc.), which could moderate the strength of the relationships presented here. Although these analyses cannot prove causation, they demonstrate that loneliness was related to increased psychological difficulties.

The pattern of results is also not fully explained by the pandemic and may be likely explained by previously identified risk factors for loneliness, such as social isolation, exacerbated by the pandemic. The first wave of this study was collected when isolation and infection rates were high worldwide. Most states had issued mandatory stay-at-home orders at Wave 1 [109]. By the second wave, all US stay-at-home orders had been lifted, and the COVID vaccination was increasingly available, making social distancing and physical isolation less likely. Our findings match previous research that risk factors for loneliness (e.g., being female, being a younger adult, physical isolation) have been nearly identical before and after the pandemic [5].

Parent support was more strongly related to decreased loneliness than either friend or SO support. Future research should examine whether the differential support that various ethnic groups rely on shifts in a pandemic. White and Asian emerging adults have been found to rate their friends higher on global social support than parents, whereas there were no differences between friends and family support in the three Latino groups [96]. It is possible that as job and living arrangements become more unstable during the pandemic, White and Asian youth may also rely more globally on the support of their parents. Future research should also examine the type of support each relationship provides and whether these support dimensions shift during a pandemic. For example, do patterns shift such that parents may provide increasing levels of companionship and intimacy, as quality time with friends may be impeded? Or does affection and instrumental help become more critical to the emerging adult in times of crisis [96]?

The current study offers evidence of the concurrent and longitudinal relationships between social support, loneliness, and mental health in US emerging adults during the COVID-19 pandemic. The results highlight the need for continued research as future pandemics are likely. As the everyday lives of emerging adults shift in the immediate and distant future in response to COVID-related changes, their loneliness will likely fluctuate as well. Researchers, practitioners, and policymakers should use results like the current study to look for ways to reduce loneliness in emerging adults and prevent other adverse health outcomes (e.g., sleep difficulties and depression). Specifically, more research is needed to determine the most effective ways to mitigate the negative impact of loneliness on the health of emerging adults, especially in times of heightened stress. This study provided further evidence that social support is critical for reducing loneliness and mental health problems; it is imperative to consider the influence of social support and loneliness on mental health and how these experiences of emerging adults during difficult times can be used to improve their health and well-being even as the pandemic subsides.

## Figures and Tables

**Table 1:** The direct effects of social support on mental health.

Direct Effect	Estimate	*SE*	*z*	*p*
	Depression			
Significant Other Support	−0.04	0.03	−1.46	0.14
Family Support	−0.08	0.02	−3.69	<0.001
Friend Support	0.03	0.03	1.31	0.19
	CPTSS			
Significant Other Support	−0.07	0.05	−1.34	0.18
Family Support	−0.01	0.05	−0.17	0.86
Friend Support	0.11	0.05	2.14	0.03
	Perceived Stress			
Significant Other Support	−0.05	0.03	−1.50	0.14
Family Support	−0.13	0.03	−4.44	<0.001
Friend Support	0.08	0.03	2.36	0.02
	Sleep Problems			
Significant Other Support	0.03	0.03	0.92	0.36
Family Support	0.01	0.03	0.43	0.67
Friend Support	0.04	0.03	1.46	0.15

**Table 2:** The indirect effects of social support via loneliness on mental health.

Indirect Effect	Estimate	*SE*	*z*	*p*
	Perceived Stress			
Family Support → Loneliness	−0.08	0.02	−5.14	<0.001
Friend Support → Loneliness	−0.05	0.01	−3.26	0.001
Significant Other Support → Loneliness	−0.08	0.02	−3.42	<0.001
	Depression			
Family Support → Loneliness	−0.07	0.01	−5.27	<0.001
Friend Support → Loneliness	−0.05	0.01	−3.26	0.001
Significant Other Support → Loneliness	−0.05	0.01	−3.45	<0.001
	CPTSS			
Family Support → Loneliness	−0.04	0.01	−2.88	0.004
Friend Support → Loneliness	−0.03	0.01	−2.37	0.018
Significant Other Support → Loneliness	−0.03	0.01	−2.45	0.014
	Sleep Problems			
Family Support → Loneliness	−0.05	0.01	−4.61	<0.001
Friend Support → Loneliness	−0.03	0.01	−3.08	0.002
Significant Other Support → Loneliness	−0.04	0.01	−3.25	0.001

**Table 3:** Cross-lagged relationships: Social support and loneliness in Study 2.

Variable	Estimate	*SE*	STD.ALL	*z*	*p*
	Family Support				
Time 2 Loneliness					
Loneliness	0.62	0.05	0.62	11.72	<0.001
Family Support	−0.06	0.03	−0.12	−2.39	0.020
Time 2 Family Support					
Loneliness	−0.13	0.10	−0.07	−1.37	0.17
Family Support	0.62	0.05	0.66	12.28	<0.001
Covariances (Time 2)					
Loneliness—Family Support	−0.09	0.02	−0.28	−4.23	<0.001
	Friend Support				
Time 2 Loneliness					
Loneliness	0.66	0.05	0.67	12.90	<0.001
Friend Support	−0.02	0.03	−0.04	−0.69	0.49
Time 2 Friend Support					
Loneliness	−0.12	0.10	−0.06	−1.16	0.25
Friend Support	0.60	0.06	0.58	10.41	<0.001
Covariances (Time 2)					
Loneliness—Friend Support	−0.11	0.02	−0.30	−4.56	<0.001
	Significant Other Support				
Time 2 Loneliness					
Loneliness	0.63	0.05	0.63	12.41	<0.001
Significant Other Support	−0.06	0.03	−0.12	−2.23	0.026
Time 2 Significant Other Support					
Loneliness	−0.14	0.10	−0.07	−1.33	0.18
Significant Other Support	0.60	0.06	0.59	11.01	<0.001
Covariances (Time 2)					
Loneliness—Significant Other Support	−0.13	0.03	−0.36	−5.27	<0.001

**Table 4:** Cross-lagged relationships: Loneliness and mental health in Study 2.

Variable	Estimate	*SE*	β	*z*	*p*
	Depression				
Time 2 Loneliness					
Loneliness	0.60	0.06	0.61	10.85	<0.001
Depression	0.15	0.06	0.14	2.39	0.02
Time 2 Depression					
Loneliness	0.18	0.06	0.18	3.27	0.001
Depression	0.59	0.06	0.55	9.48	<0.001
Covariances (Time 2)					
Loneliness—Depression	0.08	0.01	0.42	6.11	<0.001
	PTSD Symptoms				
Time 2 Loneliness					
Loneliness	0.65	0.05	0.66	13.73	<0.001
PTSS	0.06	0.03	0.08	1.72	0.09
Time 2 CPTSS					
Loneliness	0.22	0.08	0.15	2.78	0.005
CPTSS	0.51	0.05	0.52	9.62	<0.001
Covariances (Time 2)					
Loneliness—CPTSS	0.04	0.02	0.14	2.16	0.04
	Perceived Stress				
Time 2 Loneliness					
Loneliness	0.64	0.05	0.64	11.74	<0.001
Stress	0.06	0.05	0.08	1.34	0.18
Time 2 Stress					
Loneliness	0.23	0.07	0.19	3.29	0.001
Stress	0.55	0.06	0.53	9.06	<0.001
Covariances (Time 2)					
Loneliness—Stress	0.09	0.02	0.36	5.31	<0.001
	Sleep Difficulty				
Time 2 Loneliness					
Loneliness	0.67	0.05	0.67	13.97	<0.001
Sleep Difficulty	0.05	0.05	0.05	1.09	0.28
Time 2 Sleep Difficulty					
Loneliness	0.19	0.05	0.20	4.31	<0.001
Sleep Difficulty	0.56	0.05	0.57	11.70	<0.001
Covariances (Time 2)					
Loneliness—Sleep Difficulty	0.03	0.01	0.15	2.26	0.025

## Data Availability

The data presented in this study are available on request from the corresponding author due to IRB restrictions.
